# Endovascular Simulator Training and Shadowing in Interventional Radiology: A Comparison of Two Teaching Methods in the Curricular Training of Medical Students

**DOI:** 10.1007/s00270-024-03863-1

**Published:** 2024-10-10

**Authors:** Simon Hilleke, Richard Wiener, Anne Frisch, Michael Scheel

**Affiliations:** 1 Department of Neuroradiology, Charité – Universitätsmedizin Berlin, corporate member of Freie Universität Berlin and Humboldt-Universität zu Berlin, Charitéplatz 1, 10117 Berlin, Germany; 2 Department of Diagnostic and Interventional Radiology, Vivantes Klinikum im Friedrichshain, Landsberger Allee 49, 10249 Berlin, Germany; 3 Department of Radiology, Charité – Universitätsmedizin Berlin, corporate member of Freie Universität Berlin and Humboldt-Universität zu Berlin, Augustenburger Platz 1, 13353 Berlin, Germany

**Keywords:** Interventional radiology, Simulator training, Shadowing, Seldinger technique, Aortography, Medical education, Medical students, Teaching sequence

## Abstract

**Purpose:**

To identify the impact of endovascular simulator training and shadowing in interventional radiology on medical students’ self-assessed IR knowledge. Moreover, the sequence of the teaching methods and its influence on the self-assessed IR knowledge is investigated.

**Materials and Methods:**

A total of 19 fourth-year medical students participated in this study. Eleven students completed shadowing live cases first and endovascular simulator training the following day. Eight students completed the teaching in reversed order. Questionnaires were completed before and after each teaching method. The students assessed their knowledge of instruments and materials, steps of the Seldinger technique, and aortography on a Likert scale (1 = "I do not agree at all," 5 = "I fully agree").

**Results:**

After simulator training, the students stated a significant increase in perceived knowledge compared with baseline (*p* < 0.001). Shadowing led to a significant improvement regarding the items “knowledge of instruments and materials” (3.2 vs. 3.8, *p* = 0.008) and “steps of the Seldinger technique” (3.7 vs. 3.9, *p* = 0.046). Self-assessed knowledge after simulator training increased significantly more regarding Seldinger technique compared with shadowing (+ 1.2 vs. + 0.2, *p* < 0.001). Simulator training before shadowing was significantly more effective regarding the increase in “knowledge of the steps of aortography” compared with the reverse sequence (+ 2.0 vs. + 0.9, *p* = 0.041).

**Conclusion:**

Endovascular simulator training and shadowing are both feasible tools to improve medical students’ perceived knowledge of interventional radiology. When organizing teaching, simulator training before shadowing can have a positive impact on self-assessed knowledge.

**Graphical Abstract:**

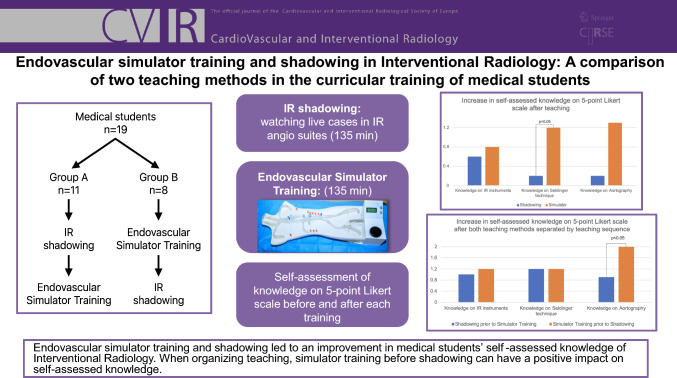

**Supplementary Information:**

The online version contains supplementary material available at 10.1007/s00270-024-03863-1.

## Introduction

Lacking integration of IR into medical school curricula and insufficient exposure to endovascular activities led to medical students complaining about deficient IR knowledge [1–4]. The curricular under-representation of IR confronts teachers with few opportunities to teach IR-related content, which makes effective teaching in IR important. Endovascular simulator training has been identified to be an effective method for increasing IR knowledge among medical students [4]. Shadowing is another common method to provide medical students with insights into specialties. In medicine, it consists of students or trainees observing a physician’s professional activities [5]. Although shadowing is a common teaching method in medicine, its effects on medical students in IR have not yet been studied. Our study aims to determine the effect of structured clinical-practical IR training consisting of shadowing in IR angio suites and endovascular simulator training. More specifically, we tried to answer the questions of whether endovascular simulator training leads to a greater increase in self-assessed IR knowledge among medical students compared with shadowing and in which implemented sequence the two teaching methods lead to a greater increase in self-assessed IR knowledge.

## Methods

### Study Design

Our study was conducted during a 14-day elective module on radiology among fourth-year medical students of which 3 days were dedicated to IR. Data were collected in two cohorts of medical students in two consecutive semesters at one IR department. A preparatory day covered theoretical content on IR conveyed in seminars and lectures and was attended by all participants together. On the following 2 days, all students completed endovascular simulator-based training and shadowing. For the practical units of the course, the students were divided into two groups and taught in a crossover design. One group (*Shadowing-Simulator*) completed the shadowing on course day one and the endovascular simulator training on course day two. The other group (*Simulator-Shadowing*) underwent the teaching in reversed order. The duration for shadowing and endovascular simulator-based training was 135 min each and took place simultaneously. Pre- and post-questionnaires were conducted before and after each course day.

## Course Content

The course was conducted by (neuro) interventional radiologists from the Department of Radiology at a large public medical school in Germany. Shadowing consisted of the group experiencing daily elective patient care as live case scenario during which the students were allowed to ask questions about the procedure, techniques, and complications. Endovascular simulator-based training was executed using a simulator that consists of a transparent, 3D-printed aortic tree and its large branches. For the training, a tube system is filled with water and a pump creates physiological pressure and flow conditions. The endovascular simulator-based training consisted of:

Arterial puncture and probing using Seldinger technique, catheter navigation with different catheters and probing of the supra-aortic vessels using vertebral catheter and Sim-2 catheter as well as probing of the Truncus coeliacus using Cobra catheter.

## Questionnaire Design

The questionnaire was designed in cooperation with experienced (neuro) interventional radiologists. The questionnaire was filled out anonymously and documented demographics and IR-specific knowledge on a 5-point Likert scale. The questionnaire is provided in the online supplement.

## Statistical Evaluation

The statistical analysis was conducted using IBM^®^ SPSS Statistics V 29.0.0.0. To assess the effect of each teaching method, the results of pre- and post-questionnaires were evaluated for both groups combined, respectively. The significance of the sequence of teaching methods was analyzed by separation of the two groups.

**Fig. 1:**
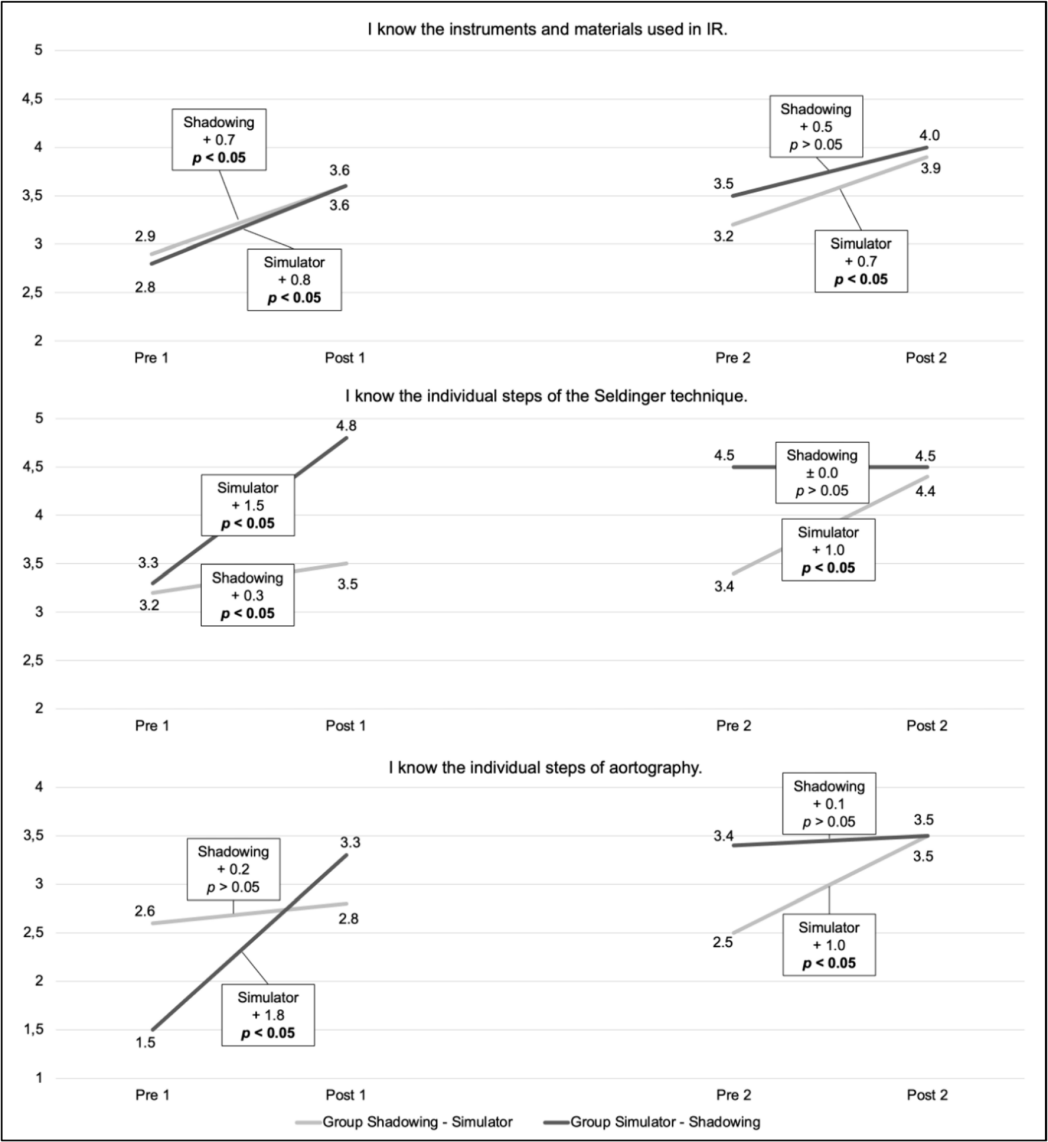
Learning curve throughout the two course days represented by the results of self-assessed knowledge on a 5-point Likert scale before and after undertaking each teaching method and significance levels. Pre-1 and post-1 represent the first and pre-2 and post-2 represent the second course day. The groups and their sequence of teaching methods are indicated in light gray (shadowing first) and dark gray (simulator training first)

Changes within a group and the effect of a singular teaching method were evaluated using the Wilcoxon test for connected samples. Comparison of the groups regarding the sequence was executed using the Mann–Whitney U test for independent samples. To evaluate the difference between the employed teaching methods, we used a mixed-effects model with random intercepts. A *p*-value < 0.05 was defined as significant. Figure 1 was created using Microsoft^®^ Excel version 16.73.

## Results

### Demographics

A total of 35 students took part in the elective module in consecutive semesters (19 students in the first semester and 16 students in the following semester). Of these in total, 19 students (mean age in years: 26 [SD 7.5]; 14 female, 5 male) were included in the study, which participated in shadowing and simulator-based training as well as completed all four questionnaires. Group *Shadowing-Simulator* consisted of eleven students (mean age in years: 25 SD [4.6]; eight female, three male) and Group *Simulator-Shadowing* of eight (mean age in years: 28 SD [10.4]; six female, two male). One person (Group *Shadowing-Simulator*) indicated experience in radiology (clinical elective).

## Shadowing and Endovascular Simulator-Based Training

The results of the pre- and post-questionnaires for the individual teaching methods as well as the comparison of the two teaching methods are presented in Table 1.

**Table 1:** Change in mean value of self-assessed knowledge (on a 5-point Likert scale) sorted by applied teaching method and the comparison of the two teaching methods

	Shadowing pre vs. post	Simulator training. pre versus post	Shadowing versus Simulator training
I know the instruments and materials used in IR	**3.2 versus 3.8** ***(p* = 0.008*)***	**3.0 versus 3.8** ***(p* < 0.001*)***	+ 0.6 versus + 0.8 *(p* = 0.892*)*
I know the individual steps of the Seldinger technique	**3.7 versus 3.9** ***(p* = 0.046*)***	**3.3 versus 4.5** ***(p* < 0.001*)***	** + 0.2 versus + 1.2** ***(p* < 0.001*)***
I know the individual steps of aortography	2.9 versus 3.1 *(p* = 0.454*)*	**2.1 versus 3.4** ***(p* < 0.001*)***	+ 0.2 versus 1.3 *(p* = 0.052*)*

Significance levels are stated in brackets. Significant changes are presented in bold

## Increase in Self-Assessed Knowledge Depending on the Teaching Format Sequence

The results of the pre- and post-questionnaires for the individual teaching sequences, as well as the comparison of the groups’ overall increase in self-assessed knowledge, are presented in Table 2. Moreover, the learning curve represented by the progression of self-assessed knowledge for the individual groups throughout the two course days is portrayed in Fig. 1.

**Table 2:** Change in mean value of self-assessed knowledge (on a 5-point Likert scale) sorted by applied teaching sequence and comparison of the groups’ increase in self-assessed knowledge

	Group Shadowing-Simulator	Group Simulator-Shadowing	Overall increase
	pre-shadowing vs. post-simulator training	pre-simulator training vs. post-shadowing	Group Shadowing-Simulator vs. Group Simulator-Shadowing
I know the instruments and materials used in IR	**2.9 versus 3.9** ***(p* = 0.009*)***	**2.8 versus 4.0** ***(p* = 0.015*)***	+ 1.0 versus + 1.2 *(p* = 0.545*)*
I know the individual steps of the Seldinger technique	**3.2 versus 4.4** ***(p* = 0.016*)***	**3.3 versus 4.5** ***(p* = 0.014*)***	+ 1.2 versus + 1.2 *(p* = 0.904*)*
I know the individual steps of aortography	**2.6 versus 3.5** ***(p* = 0.023*)***	**1.5 versus 3.5** ***(p* = 0.011*)***	** + 0.9 **versus** + 2.0** ***(p* = 0.041*)***

Significance levels are stated in brackets. Significant changes are presented in bold

## Discussion

The results indicate that medical students benefit from endovascular simulator training as well as shadowing and increase their self-assessed IR knowledge. Simulator training led to a significant increase in perceived knowledge in the students' self-assessment regarding all items of basic IR skills. Shadowing showed a significant increase in self-assessed knowledge of instruments and Seldinger technique. The gain in self-assessed knowledge about the steps of the Seldinger technique was significantly greater after simulator-based training compared with shadowing.

Several studies investigated medical students’ receptiveness for IR revealing that curricular under-representation resulted in lack of knowledge and interest [1–3, 6, 7]. In a systematic review from 2019, Emin et al. examined the international representation of IR in medical schools and described poor IR knowledge in more than half of the students [6]. In response to poor knowledge and under-representation in medical school curricula, the *Cardiovascular and Interventional Radiological Society of Europe* (CIRSE) published the first "Curriculum of Interventional Radiology for Medical Students" in its second edition in 2019 [8].

Referring to the CIRSE publication, Shaikh et al. studied the impact of a ten-hour lecture series based on the CIRSE curriculum on medical students. The effects of the intervention were tested through a quiz on IR before and after the 2-day theoretical teaching. After the lectures only 17% rated their IR knowledge as poor or non-existent compared with other disciplines after the teaching format compared with 62% before the intervention [9].

In contrast to theoretical teaching, other studies looked at the effects of hands-on training on students [4, 10–12]. In 2020, Stoehr et al. compared the effect of theoretical teaching with endovascular simulation-based training on fourth-year medical students regarding the change in IR knowledge and enthusiasm. Students participated in a 90-min training using an endovascular simulator. Knowledge and interest in IR were documented on a Likert scale. Both teaching methods led to a significant increase, whereby the endovascular simulator training was significantly more effective compared with the theoretical seminar. Stoehr et al. summarized the increase in knowledge as "knowledge of endovascular techniques" [4]. In comparison, in our study, we tried to obtain more precise information about which knowledge the simulator training conducted. In this way, we were able to shed more light on the effects on knowledge regarding instrument and material, the steps of the Seldinger technique and the steps of aortography, as basics of IR.

Some studies outside IR have already dealt with the effects of shadowing on medical students [5, 13, 14]. Evans et al. investigated the impact of a 5-day program mostly consisting of shadowing on the clinical skills of newly qualified doctors. Participants underwent pre- and post-program assessment using the Objective Structured Clinical Examination (OSCE) format, which tested basic clinical skills. The program led to a significant improvement in clinical skills and an improvement in confidence [15].

Teaching IR to medical students can be challenging considering curricular under-representation and limited teaching options. The analysis of the individual interventions’ sequence can be beneficial to the organization of training and an efficient transfer of knowledge. We found that greater overall knowledge gains were achieved in the group that received simulation-based training before shadowing. However, only regarding the steps of aortography a significant difference in gain of the self-assessed knowledge compared with group with an inverted teaching method sequence was found.

The small number of participants is one of the main limitations of our study. Moreover, in contrast to simulator training, shadowing elective live cases does not provide a reproductive structure. Self-assessment on Likert scale to measure the medical students’ knowledge is an established way to get an impression of a teaching effect [8, 14]. Yet response bias must be considered when using a questionnaire-based approach. It can lead to respondents answering questions untruthfully because they think they are expected to answer in a certain way. We used an anonymous study design to counteract this pitfall. For objective quantification of knowledge, other forms of evaluation such as a pre- and post-quiz, evaluation of performance on the endovascular simulator through an expert and measurement of time practicing on the simulator should be implemented in further studies. Moreover, we only analyzed the practical parts of the course and not the theoretical seminar on the 1st day.

## Conclusion

Both endovascular simulator training and shadowing have positive effects on the medical students’ self-assessed IR knowledge. In some cases, medical students seem to benefit more from hands-on teaching on an endovascular simulator compared with shadowing. In the organization of IR teaching in medical schools with few teaching opportunities and limited teaching time, simulator training before shadowing can be advantageous. Further research with a larger sample and objective verification of knowledge is needed.

## Supplementary Information

Below is the link to the electronic supplementary material.

Supplementary file1 (pdf 101 KB)
