# Supplementary material for: Endovascular Simulator Training and Shadowing in Interventional Radiology: A Comparison of Two Teaching Methods in the Curricular Training of Medical Students
Source: Cardiovasc Intervent Radiol. 2024 Oct 10;47(11):1540–6. doi: 10.1007/s00270-024-03863-1 (PMC11541414; doi:10.1007/s00270-024-03863-1)
Supplement: Supplementary file 1 — Supplementary file1 (pdf 101 KB) [file 270_2024_3863_MOESM1_ESM.pdf]

Pre- and post-questionnaire

ID					
Age (in years)					
Sex	Female ◯	Male ◯	Other ◯		
Training in Radiology	None ◯	Clinical elective ◯	Training in Radiology (e.g. MTRA) ◯	Other (please specify):	

	1 (I strongly disagree)	2 (I tend to disagree)	3 (I am not sure)	4 (I tend to agree)	5 (I strongly agree)
I know the instruments and materials used in IR.	◯	◯	◯	◯	◯
I know the individual steps of the Seldinger technique.	◯	◯	◯	◯	◯
I know the individual steps of aortography.	◯	◯	◯	◯	◯
